# Does the rate of orthodontic tooth movement change during the estrus cycle? A systematic review based on animal studies

**DOI:** 10.1186/s12903-021-01875-8

**Published:** 2021-10-14

**Authors:** Noura Saeed Sultan Almidfa, Athanasios E. Athanasiou, Miltiadis A. Makrygiannakis, Eleftherios G. Kaklamanos

**Affiliations:** 1 Hamdan Bin Mohammed College of Dental Medicine (HBMCDM), Mohammed Bin Rashid University of Medicine and Health Sciences (MBRU), Building 34, Dubai Healthcare City, Dubai, United Arab Emirates; 2 Dubai Health Authority, Dubai, United Arab Emirates; 3 Department of Dentistry, European University Cyprus, Nicosia, Cyprus; 4 Department of Orthodontics, School of Dentistry, National and Kapodistrian University of Athens, Athens, Greece

**Keywords:** Estrus, Menstrual cycle, Tooth movement, Orthodontics

## Abstract

**Background:**

As the fluctuation of sex hormone levels in menstruating women results in periodical effects in bone metabolism, understanding the implications for tooth movement could be of benefit to the orthodontist. This type of research presents practical and ethical problems in humans, but animal models could provide useful information. Our objective was to systematically investigate the available evidence on the question whether the rate of orthodontic tooth movement varies between the different stages of the estrus cycle in animals.

**Methods:**

Unrestricted searches in 7 databases and manual searching of the reference lists in relevant studies were performed up to February 2021 (Medline [PubMed], CENTRAL [Cochrane Library; includes records from Embase, CINAHL, ClinicalTrials.gov, WHO's ICTRP, KoreaMed, Cochrane Review Groups’ Specialized Registers, and records identified by handsearching], Cochrane Database of Systematic Reviews [Cochrane Library], Scopus, Web of Knowledge [including Web of Science Core Collection, KCI Korean Journal Database, Russian Science Citation Index, SciELO Citation Index and Zoological Record], Arab World Research Source [EBSCO] and ProQuest Dissertation and Theses [ProQuest]). Our search focused on prospective controlled animal studies, whose samples included female subjects of any species that were quantitatively comparing the amount of tooth movement in the different stages of the estrus cycle. Following study retrieval and selection, relevant data was extracted, and the risk of bias was assessed using the SYRCLE’s Risk of Bias Tool.

**Results:**

From the finally assessed records, 3 studies met the inclusion criteria. Two of the studies experimented on Wistar rats, whereas the other on cats. Tooth movement was induced by expansion or coil springs. The rate of orthodontic tooth movement was increased during the stages of the estrus cycle when oestrogen and/or progesterone levels were lower. The risk of bias in the retrieved studies was assessed to be unclear.

**Conclusion:**

Hormonal changes during the estrus cycle may affect the rate of orthodontic tooth movement. Although these animal experiment results should be approached cautiously regarding their translational potential, it could be useful to consider the possible impact of these physiological changes in the clinical setting until more information becomes available.

*Registration*: PROSPERO (CRD42021158069).

**Supplementary Information:**

The online version contains supplementary material available at 10.1186/s12903-021-01875-8.

## Background

The menstrual cycle in women constitutes a sequence of events characterized by periodic and repeated hormonal fluctuations that prepare the female body for a potential pregnancy [1]. Estradiol levels are at baseline during menses, then slowly increase and reach a peak a day or two before ovulation. After ovulation, estradiol concentrations drop abruptly and during most of the luteal phase, the production of estradiol is maintained at low levels, before decreasing more and reaching the lowest concentrations during menstruation. Progesterone is mostly produced after ovulation during the luteal phase of the menstrual cycle [1]. Both estradiol and progesterone have been shown to affect bone turnover [2]. Zittermann et al. observed that the physiological menstrual cycle in females is associated with corresponding periodical changes in bone turnover, closely related to the fluctuations in serum estradiol [3].

Orthodontic tooth movement involves intracellular pathways, intercellular physiological signaling processes, as well as interactions between cells and the extracellular environment that are regulated by hormones, growth factors and cytokines [4]. Information from studies conducted in female experimental animals have suggested that bone metabolism alterations occurring during pregnancy and lactation, as well as the osteoporotic changes following ovariectomy, may have an impact on the rate of tooth movement [5, 6]. Furthermore, it could be hypothesized that the endocrine controls that govern the menstrual cycle may influence bone resorption and apposition under the effect of orthodontic forces as well. Xu et al. [7] suggested orthodontic treatment to be scheduled according to the menstrual cycle and orthodontic forces to be applied after ovulation, to potentially increase the speed of tooth movement and shorten orthodontic treatment duration.

Thus, understanding the impact of the physiological mechanisms that determine the menstrual cycle’s timing of the events on orthodontic tooth movement and considering the possible implications may be of benefit. However, this type of research presents significant limitations. During history, ethical and practical considerations as well as social bans have prevented experimental studies on human subjects [8]. Animals have been used extensively in studies of human reproduction research [9, 10].

### Objective

The aim of the present study is to systematically investigate and appraise the quality of the available evidence on the question whether the rate of orthodontic tooth movement varies between the different stages of the estrus cycle in animals.

## Methods

### Protocol development

The present review was based on a protocol developed, registered, carried out and reported following relevant methodological guidelines (PROSPERO: CRD42021158069) [11–14]. As the present study is a systematic review, ethical approval was not required.

### Eligibility criteria

The Participants, Intervention, Comparator and Outcomes domains were used to describe the eligibility criteria (PICO) (Additional file 1: Table S1). We looked for prospective experimental controlled studies on healthy female animals (Participants) evaluating the rate of tooth movement (Outcomes) in the different stages of the estrus cycle (Comparator). All types of orthodontic interventions to induce movement of teeth were considered (Intervention), and the studies had to report on the amount of tooth movement either during or after the cessation of orthodontic forces. Tooth movement could be measured in various ways (with calipers, feeler gauges, etc. directly or from plaster models; from histological cuts directly on the optical microscope or from digital photos; radiographs of any kind i.e., lateral cephalometric radiographs, Cone Beam CT, micro-CT, etc.). We excluded studies on male animals as well as female animals under medication, with dietary deficiencies or ovariectomy. Studies involving animals with additional clinical interventions such as tooth extraction, etc. were also excluded, as well as studies presenting qualitative assessments. Finally, we did not consider human, in vitro, ex-vivo or in silico studies; non-comparative studies, reviews, systematic reviews, meta-analyses, and studies with fewer than 5 animals per group analyzed, as per relevant methodological guidelines regarding the consideration of degrees of freedom for treatment comparisons [15, 16].

### Information sources and search strategy

One author (EGK) developed the detailed search strategies for each of the databases that were searched until February 17th, 2021 (Medline [PubMed], CENTRAL [Cochrane Library; includes records from Embase, CINAHL, ClinicalTrials.gov, WHO's ICTRP, KoreaMed, Cochrane Review Groups’ Specialized Registers, and records identified by handsearching], Cochrane Database of Systematic Reviews [Cochrane Library], Scopus, Web of Knowledge [including Web of Science Core Collection, KCI Korean Journal Database, Russian Science Citation Index, SciELO Citation Index and Zoological Record], Arab World Research Source [EBSCO] and ProQuest Dissertation and Theses [ProQuest]) (Additional file 1: Table S2). We did not impose any restrictions on the language or date of publication. Duplicates were removed using EndNote's duplicate identification strategy (EndNote X9™, Clarivate™, Philadelphia, PA, USA) and then manually by EGK. We also manually searched the reference lists in relevant article to identify additional studies (NSSA and MAM).

### Selection process, data collection process and data items

Two authors (NSSA and MAM) assessed the retrieved records for inclusion independently. They were not blinded to the identity of the authors, their institution, or the results of the research. If the abstract was unclear, the full paper was accessed to determine the eligibility for inclusion.

From the finally eligible studies, the following information was extracted by NSSA and MAM independently in predetermined forms when available: bibliographic information, study design and eligibility; type of experimental groups; number of animals in each group and sample size calculation; age and weight of animals; orthodontic mechanics; measurement of outcome details and reliability assessment. Results were to be extracted and categorized separately for each species or type of mechanics used, since differences can be expected [17]. If clarifications were needed regarding the published data, or additional material was required, then attempts to contact the corresponding authors through email were made.

### Study risk of bias assessment

NSSA and MAM assessed the risk of bias in the included studies, independently and in duplicate, during the data extraction process, using the SYRCLE’s risk of bias tool [18]. Assessments were subsequently entered into the Risk-of-bias VISualization (robvis) web application [19]. In all the processes, disagreements were settled by discussion with AEA; following the relevant suggestions, kappa statistics were not calculated [13].

### Effect measures, synthesis methods, certainty assessment and additional analyses

Though a synthesis of the results was planned, it was not, in the end, carried out due to methodological diversity [13]. Due to inadequate information, analyses for “small-study effects” and publication bias, as well as subgroup analyses were not performed [13]. Finally, despite the lack of extensive information, the quality of available evidence regarding the differences in the rate of tooth movement between the various stages of the estrus cycle was assessed with the Grades of Recommendation, Assessment, Development, and Evaluation to adopt a structured and transparent approach in formulating an interpretation of the evidence [20].

## Results

### Study selection

**Fig. 1:**
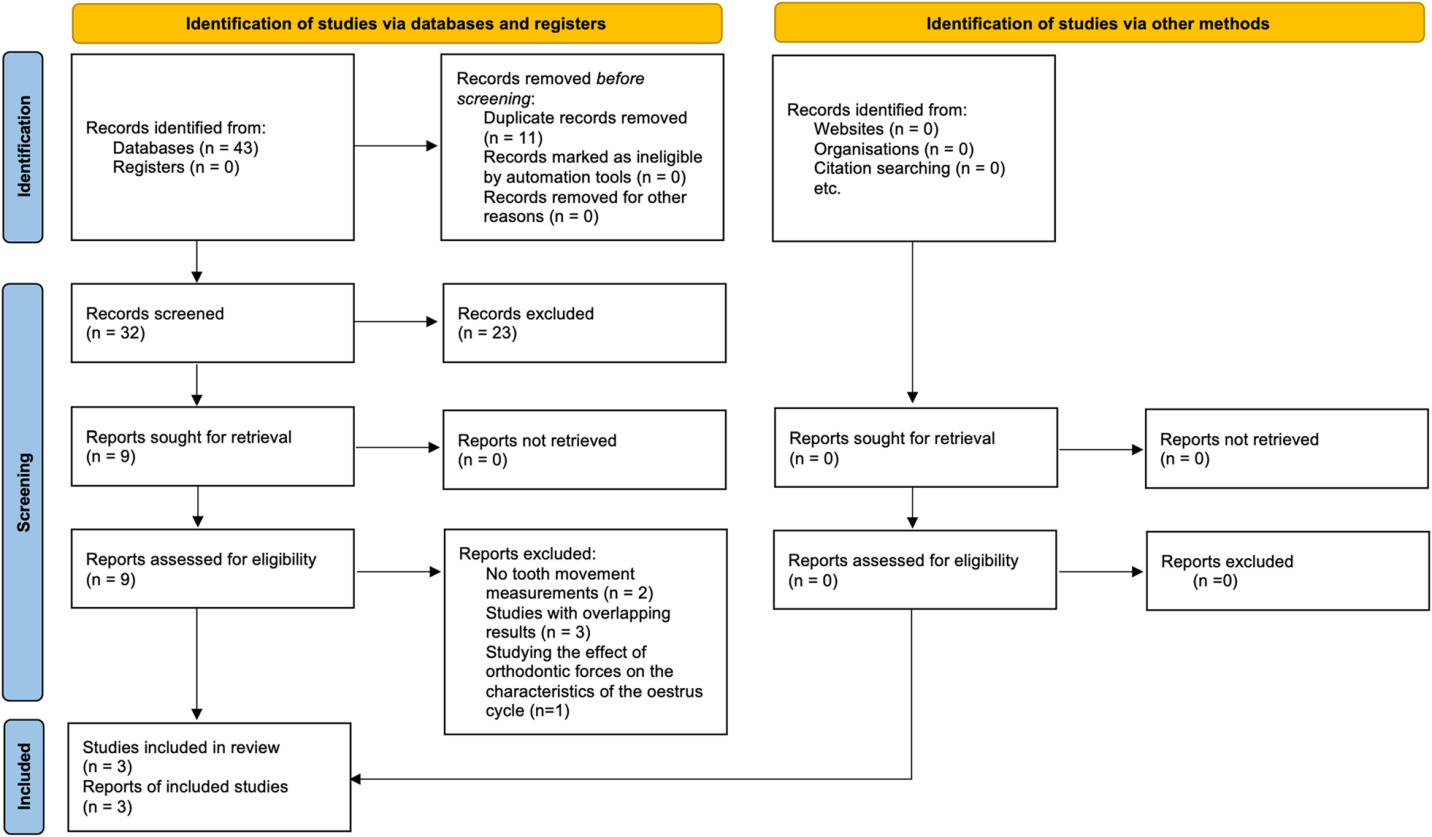
PRISMA 2020 flow diagram

Following database searches, we collected 43 records. 32 remained after the exclusion of duplicates and further 23 were excluded based on their title and abstract. Nine records were retrieved in full text and 6 were excluded for the following reasons: no tooth movement measurements [21, 22]; including overlapping information [23–25]; studying the effect of orthodontic forces on the characteristics of the estrous cycle [26]. Finally, three full text reports were included in the systematic review [27–29] (Fig. 1).

### Study characteristics

The retrieved studies were published between 2002 and 2012 and investigated the influence of estrus cycle on the rate of orthodontic tooth movement during force application. Two types of animals were used: cats [29] and Wistar rats [27, 28]. An expansion spring between the upper first molars was used by Haruyama et al. [27]. The other two studies used NiTi coil springs to retract the canine [29] or medialize the first molar [28]. The forces exerted ranged between 13 and 80 g. The rate of tooth movement was measured from plaster models [28]; from silicone impressions [29] and from tracings of the occlusal surface of maxillary casts [27]. No sample size calculations were performed and only Haruyama et al. assessed the error of the method [27].

In the investigations on rats [27, 28], animals in the estrus, metestrus, diestrus, and proestrus groups, defined by vaginal smears, received a force for 2 days during each estrous cycle from late proestrus, late estrus, late metestrus, and late diestrus, respectively. Subsequently, no force was applied for the remaining days in each estrous cycle. The animals were examined for 5 consecutive estrous cycles and received the force 5 times for 2 days in each estrous cycle. Tooth movement measurement was performed after the removal of the orthodontic appliance.

In the study of Celebi et al. [29], the cats were randomly divided into estrous and anestrous. In the former group, estrous was induced by the administration of equine chorionic gonadotropin. In the anestrous group, tooth movement was performed during the nonbreeding season, confirmed by blood estradiol levels being at basal value. Tooth movement was measured on plaster models, produced by impressions taken on days 0, 6 and 12.

### Risk of bias in studies

**Fig. 2:**
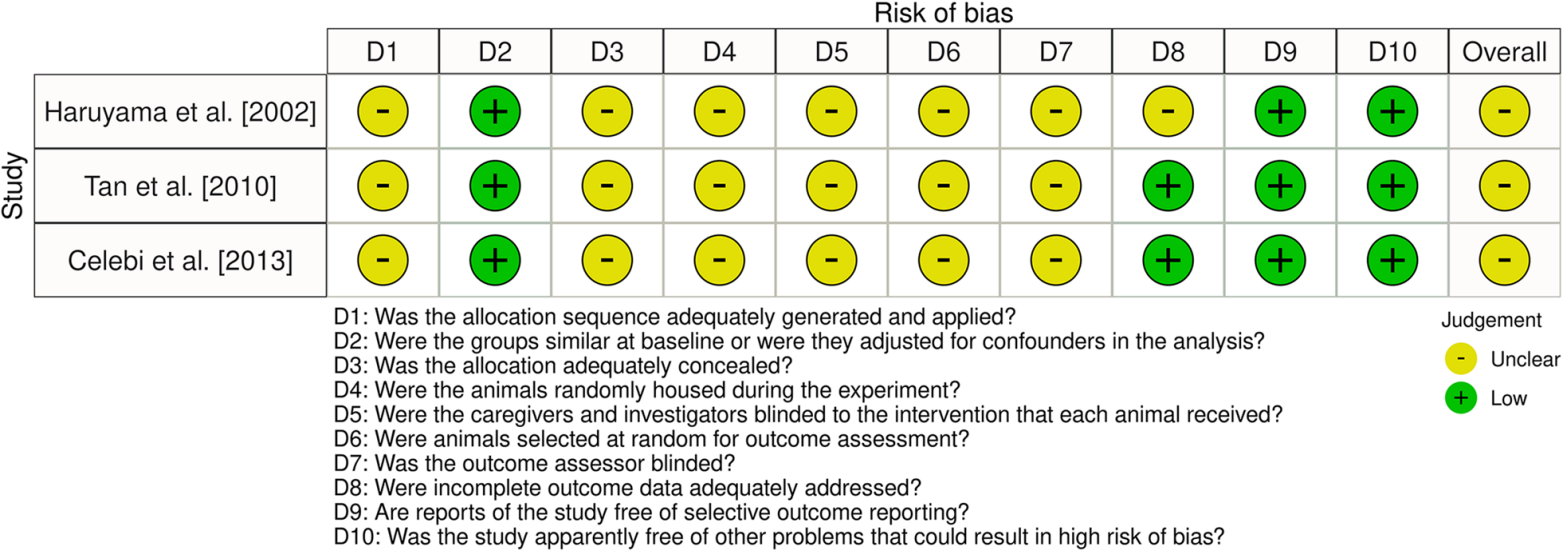
Risk of bias assessment

Figure 2 presents the summary of findings regarding risk of bias assessment. The assessed domains were found to be mostly at unclear risk of bias. The risk of bias for the domains of baseline similarity, selective outcome reporting and other problems was assessed to be low. Regarding incomplete outcome data, the risk of bias was assessed to be unclear for Haruyama et al. [27] because of the significant number of animals excluded from the final analysis.

### Effect of estrus cycle on the rate of orthodontic tooth movement

Overall, tooth movement increased in the stages of the estrus cycle when the estradiol and/or progesterone levels were lower (Table 1). Haruyama et al. [27] and Tan et al. [28] showed an increase in tooth movement in the estrus animals. In estrous rats, the estradiol and progesterone levels are expected to be at their lowest [10]. In Haruyama et al. [27] tooth movement was greater in the estrus group by 32.6% compared to the proestrus group (*p* < 0.05; Tukey–Kramer test). Tooth movement in the metestrus and diestrus group did not differ significantly. Tan et al. observed 2.10 ± 0.14 mm total tooth movement in the estrus stage, while the lowest result was for the proestrus stage (1.79 ± 0.03 mm) (*p* < 0.05; Student–Newman–Keuls test) [28]. Tooth movement in the metestrus (1.94 ± 0.04 mm) and diestrus (1.89 ± 0.06 mm) groups did not differ significantly.

**Table 1:** Characteristics of the included studies

Study	Animals and tooth movement model	Group Characteristics	Measurement Methodology	Results ^b^			
Haruyama et al. [27]	Wistar rats; 10 weeks; 136 g NiTi expansion spring between R and L FM [13 g] Force application: at each stage for 5 cycles	Proestrus Group: 8 estrus Group: 8 Metestrus Group: 8 Diestrus Group: 8 Sample size calculation: nm	Tracings of casts: callipers [at appliance removal] Method error assessment: Yes	Estrus	Metestrus	Diestrus	Proestrus
				*			
				Estrus > Proestrus			
Tan et al. [28]	Wistar rats; 3 months; 300 g NiTi CCS between Mx I and FM [20 g] Force application: at each stage for 5 cycles	Proestrus Group: 10 Estrus Group: 10 Metestrus Group: 10 Diestrus Group: 10 Sample size calculation: nm	Plaster models: callipers [at appliance removal] Method error assessment: nm	Estrus	Metestrus	Diestrus	Proestrus
				*			
				Estrus > Proestrus			
Celebi et al. [29]	Cats; 2–4 yrs NiTi CCS from mini implants to Mx C [80 g] Force application: 12d	Estrus Group: 6 ^a^ Anestrus Group: 6 Sample size calculation: nm	Silicone impressions: digital callipers [d 0,6,12] Method error assessment: nm	Estrus < Anestrus^a^			

d, days; CCS, closed coil spring; FM, first molars; L, left; Mx, maxillary; NS, non-significant; R, Right

^a^Estrus was induced by administration of 150 IU equine chorionic gonadotropin. In cats, estrus is characterized by increased estrogen levels. In the anestrus group blood estradiol levels were at basal value

^b^The asterisk denotes a statistically significant difference

Celebi et al. [29] showed slower tooth movement in the estrus group, but in cats this stage is characterized by increased estradiol levels [30] (6 days, estrus (Mean ± SD): 0.546 ± 0.055 mm; Anestrus (Mean ± SD): 0.659 ± 0.107 mm, *p* < 0.05, ANOVA; 12 days, estrus (Mean ± SD): 0.742 ± 0.058 mm; Anestrus (Mean ± SD): 0.992 ± 0.108 mm, *p* < 0.05, ANOVA). Regarding the differences in the rate of tooth movement between the various stages of the estrus cycle the quality of available evidence was considered as moderate (Additional file 1: Table S3).

## Discussion

Sex hormone levels fluctuate in menstruating women resulting in periodical effects in bone metabolism [3]. Potentially orthodontic tooth movement could be affected as well. Based on the data from the located animal studies, the amount of movement increased at the stages of the estrus cycle in which estradiol and/or progesterone levels were lower. Although information from the identified animal studies cannot be fully translated to humans and the risk of bias was mostly unclear, it could be useful not to ignore these observations, as well as consider the possible implications until more scientific information becomes available.

The human menstrual cycle is comparable to the estrus cycle of rats; not only is the maintenance mechanism of the periodic rhythm similar in both cycles, but also the control of estrogen levels [10, 31] In rats, the estrus cycle includes the following four stages: proestrus, estrous, diestrus 1 (or metestrus) and diestrus 2 (diestrus) [31]. Traditionally the estrus cycle is described to start from proestrus; however, recent descriptions start from diestrus 1 (or metestrus) and diestrus 2 (diestrus), which correspond to the follicular phase of the menstrual cycle [31]. The ovarian estrus cycle starts with a follicular phase, which is characterized by the development of follicles from oocytes in the rat ovary and is stimulated by low concentrations of follicle stimulation hormone (FSH) that are secreted from the pituitary. Moreover, during this period a gradual increase of estradiol levels is observed. This phase lasts around 2 days, the first day called diestrus 1 or metestrus, and the second day is diestrus 2 or just diestrus. Metestrus is also characterized by the activity of the corpus luteum, which produces progesterone and is cytologically characterized by nucleated and cornified cells, whereas diestrus cells consist mainly of leukocytes [10, 31, 32]. During proestrus, which in corresponds to the pre-ovulatory period, estradiol increases dramatically, triggers gonadotrophin-releasing hormone (GnRH) release and induces a surge of luteinizing hormone (LH) from the pituitary that induces ovulation. Progesterone rises a few hours before ovulation and contributes to this process. Once LH and progesterone are released into the circulation, ovulation occurs 10–12 h later. At the stage of proestrus vaginal cytology shows many non-cornified nucleated epithelial cells [10, 31]. Estrus refers to the stage when the female is sexually receptive and corresponds to the actual day of ovulation. It comes after the LH surge and ovulation, and during this period estradiol and progesterone come to baseline levels. The estrus phase usually lasts 25–27 h and cytological examination reveals 75% nucleated cells and 25% cornified cells [10, 31]. In cats, estrus is characterized by increased estradiol levels [30].

In the two studies performed on rats, the rate of movement was greater in the estrus group where estradiol and progesterone levels are expected to be at their lowest levels. Conversely, it was lower in the proestrus animals when estradiol levels are supposed to peak [10, 27, 28]. Indeed, estradiol levels varied according to the estrous cycle stage, as expected, demonstrating their peak at proestrus and the lowest concentration during estrus [27, 28]. The rate of tooth movement was inversely related to estradiol measurements [27]. Also, negative correlations were noted between estradiol and serum tartrate-resistant acid phosphatase (TRAP) activity and pyridinoline, both being markers of bone resorption [27].

The negative correlations observed between estradiol and bone resorption markers by Haruyama et al. [27] come into agreement with human studies [3, 33]. Estradiol is the most potent sex hormone, is produced in the ovaries and is responsible for the reproductive and sexual function of the females [34]. Estradiol also affects bones, as it regulates the osteoclastic activity and stimulates the osteoblastic activity, being essential to maintain adequate bone mass and mineralization [35, 36]. Inhibition of bone remodeling by estradiol results from preventing osteoclasts differentiation from marrow precursors, induction of osteoclast apoptosis and effects on the receptor activator of nuclear factor-Kappa B (RANK)/RANK ligand (RANKL)/osteoprotegerin (OPG) system [37, 38]. Moreover, estradiol contributes directly to bone preservation by exerting effects on the cells of the osteoblastic lineage [39, 40].

In the context of orthodontic treatment, ovariectomy induced estrogen deficiency has been associated with an acceleratory effect on the rate of tooth movement [6], while its administration reduces the speed of movement in osteoporotic rats [41, 42]. The rate of tooth movement is closely related to the activity of osteoclasts [43]. Estradiol may hinder movement through alpha receptors mediated mechanisms, as osteoclast numbers increase, and osteoblast numbers lower during tooth movement in ERα deficient mice [44].

Progesterone has been reported to downregulate bone resorption not only through direct effects on the osteoblasts, but also indirectly through the glucocorticoid receptors and the metalloproteinases [45]. It has also been associated with reductions in the amount of orthodontic tooth movement in experimental animals [46]. In the Haruyama et al. study [27], serum progesterone exhibited a different fluctuating pattern from estradiol, with its peak in diestrus. However, the lowest levels were measured during estrus, exactly like estradiol [27]. Serum osteocalcin showed a significant correlation with progesterone [27], indicating the dependence of bone-forming activity on progesterone concentration. Within the bone microenvironment, the progesterone receptor is expressed by both osteoblasts and osteoclasts [47–49]. Furthermore, estradiol can stimulate the progesterone receptor expression. Hence, it is possible that some of the bone effects attributed to estradiol may be partially regulated via progesterone signaling [47, 49, 50].

Another important parameter that needs to be highlighted is the fact that there was a variation in the species of the animals that were used for the experiments. Consequently, no direct comparisons can be made between studies, and we need to be cautious when extrapolating the results to human clinical scenarios. In specific, the study experimenting on rats, observed slower tooth movement in the estrus group [29]. However, in cats this stage is characterized by increased estradiol levels [30]. In this group, folliculogenesis and estrus were predictably induced by the exogenous administration of equine chorionic gonadotropin [51]. Prostaglandin E2 and interleukin-1b concentrations were significantly increased in the anestrus animals, where the greatest tooth movement rate was observed. Meanwhile, they were at the lowest point for the estrous group, where the slowest rate of tooth movement took place.

Estradiol stimulates the production of some pro-resorptive cytokines, like interleukins-1, -6, -7 and the tumour necrosis factor [52–58]. Especially serum interleukin-1β and -6, have been shown to play a principal role in bone resorption [59, 60] and to fluctuate during the menstrual cycle in humans [61, 62]. When an orthodontic force is applied, prostaglandins are expressed [63] and exogenous prostaglandin administration has been demonstrated to increase the rate of tooth movement in humans [64].

Even though the data retrieved were not extensive, some points arising from the reviewed information might be relevant to the treatment of menstruating female patients. It could be possible that active treatment could be shortened if orthodontic activations are performed after ovulation and/or during menstruation when estradiol levels are lower, as a pilot study has suggested [65]. In such cases however, in terms of mechanotherapy, patients might present increased needs for anchorage preparation or altered biomechanical systems because of the altered bone turnover [66]. Moreover, the absence of estradiol has been associated with greater root resorption following orthodontic tooth movement [42]. Although not directly studied in the material retrieved, one could also assume that retention procedures should be initiated when high levels of estradiol or progesterone are circulating. Histological and molecular investigations have suggested that the removal of orthodontic appliances might lead to instantaneous alterations in the mechanical environment, which could result in phenomena like those observed during active treatment but in the opposite direction [67].

### Strengths and limitations

For the current review we adhered to widely accepted methodological standards which counts to the strengths. All searches had no restrictions imposed, and all processes were performed in duplicate, while discussion helped to settle discrepancies. Finally, as similar investigations might encounter significant practical obstacles in human subjects, the current review summarized the available information from animal models that have been used extensively in female reproduction research [9].

There are also some limitations, arising mainly from the nature and the characteristics of the included studies and the information retrieved. It must be kept in mind that the collected information relates to animal studies and thus cannot be directly extrapolated to humans. Significant differences between rats, felids and humans exist, not only in terms of bone physiology, but also regarding the estrus and menstrual cycle characteristics [1, 10, 30, 68]. The lack of relevant research and power sample calculations were additional limitations affecting the precision of the retrieved results. The use of specific modes to induce orthodontic tooth movement decreases the generalizability of the retrieved information to human clinical scenarios. Also, several omissions in the report of the studies led to unclear conclusions regarding the risk of bias. Consequently, it cannot be clearly determined whether orthodontic tooth movement in humans will vary in the different stages of the menstrual cycle. Nevertheless, we should consider that analogous studies in human could confront limitations in practice.

### Recommendations for future research

Female individuals constitute most orthodontic patients making further standardized studies warranted [18, 69]. Besides, future investigation should simulate, as closely as it is feasible, human clinical scenarios to deepen our understanding of the relevant phenomena.

## Conclusions

Hormonal changes during the estrus cycle may affect the rate of orthodontic tooth movement in animals. Although these animal experiment results should be approached cautiously regarding their translatory potential, it could be useful to consider the possible impact of these physiological changes in the clinical setting until more information becomes available.

## Supplementary Information

**Additional file 1: Table S1**. Eligibility criteria. **Table S2**. Strategy for database search [February 17th, 2021]. **Table S3**. Quality of available evidence.

## Data Availability

The data underlying this article come from those included in the relevant published articles as well as the information shared from their corresponding authors. The later will be shared upon reasonable request to the corresponding author.

## Abbreviations

FSH: Follicle stimulation hormone
GnRH: Gonadotrophin-releasing hormone
LH: Luteinizing hormone
OPG: Osteoprotegerin
PICO: Participants, Interventions, Comparisons, Study design
PROSPERO: International prospective register of systematic reviews
RANK: Receptor activator of nuclear factor-Kappa B
RANKL: Receptor activator of nuclear factor-Kappa B ligand
SYRCLE: Systematic Review Center for Laboratory animal Experimentation
TRAP: Serum tartrate-resistant acid phosphatase
