# Supplementary material for: Does the rate of orthodontic tooth movement change during the estrus cycle? A systematic review based on animal studies
Source: BMC Oral Health. 2021 Oct 14;21:526. doi: 10.1186/s12903-021-01875-8 (PMC8515643; doi:10.1186/s12903-021-01875-8)
Supplement: Supplementary file 1 — Additional file 1: Table S1. Eligibility criteria. Table S2. Strategy for database search [February 17th, 2021]. Table S3. Quality of available evidence. [file 12903_2021_1875_MOESM1_ESM.docx]

**Supplementary Table 1.** Eligibility criteria.

| **Domain** | **Inclusion criteria** | **Exclusion criteria** |
| --- | --- | --- |
| **Participants** | • Female animal subjects of any species with active oestrus cycle. | • Male animals; ovariectomised animals; animals with co-morbidities, under medication, with dietary deficiencies; human subjects |
| **Intervention** | • All types of orthodontic interventions to induce movement of teeth. | • Other kinds of interventions, like growth modification, etc.  • Subjects undergoing orthodontic tooth movement in conjunction with other clinical interventions such as tooth extraction, etc. |
| **Comparisons** | • Animals at different stages of the oestrus cycle (e.g., oestrus, proestrus, metestrus, dioestrus, anoestrus, etc.) |  |
| **Outcomes** | • Amount of tooth movement during or after the cessation of the orthodontic forces [quantified measurements with measures of central tendency and dispersion] measured by various ways [directly or from plaster models with callipers, feeler gauges, etc.; from histological cuts directly on the optical microscope or from digital photos; radiographs of any kind i.e., lateral cephalometric radiographs, Cone Beam CT, micro-CT, etc.]. | • Not quantified measurements and qualitative assessments. |
| **Study design** | • Prospective controlled studies (according to the Scottish Intercollegiate Guidelines Network algorithm for classifying study design (available at http://www.sign.ac.uk/ assets/study_design.pdf). | • In vitro, ex-vivo or in silico studies.  • Human studies  • Reviews, systematic reviews and meta-analyses.  • Less than 5 subjects per group analysed (Mead et al., 2012; Kirkwood and Hubrecht, 2010). |

Mead R, Gilmour SG, Mead A. Statistical Principles for the Design of Experiments. Cambridge: Cambridge University Press; 2012.

Kirkwood J, Hubrecht R. The UFAW Handbook on the Care and Management of Laboratory and Other Research Animals. 8^th^ ed. Chichester: Wiley-Blackwell; 2010.

**Supplementary Table 2.** Strategy for database search [February 17^th^, 2021].

| **Database** | **Search strategy** | **Hits** |
| --- | --- | --- |
| **PubMed**  http://www.ncbi.nlm.nih.gov/pubmed | (estrus OR oestrus OR estrous OR oestrous OR anestrus OR anoestrus OR anestrous OR anoestrous OR proestrus OR proestrus OR proestrous OR proestrous OR metestrus OR metoestrus OR metestrus OR metoestrous OR diestrus OR dioestrus OR diestrous OR dioestrous OR "ovarian cycle" OR ovulation OR "luteal phase" OR "uterine cycle" OR menstruation OR menses OR menstruating OR menstrual OR amenorrhea OR "proliferative phase" OR "secretory phase") AND ("tooth movement" OR "orthodontic movement" OR "orthodontic anchorage" OR root resorption) | **13** |
| **Cochrane Central Register of Controlled Trials**  http://onlinelibrary.wiley.com/cochranelibrary/search | (estrus OR oestrus OR estrous OR oestrous OR anestrus OR anoestrus OR anestrous OR anoestrous OR proestrus OR proestrus OR proestrous OR proestrous OR metestrus OR metoestrus OR metestrus OR metoestrous OR diestrus OR dioestrus OR diestrous OR dioestrous OR "ovarian cycle" OR ovulation OR "luteal phase" OR "uterine cycle" OR menstruation OR menses OR menstruating OR menstrual OR amenorrhea OR "proliferative phase" OR "secretory phase") AND ("tooth movement" OR "orthodontic movement" OR "orthodontic anchorage" OR root resorption) in Title Abstract Keyword - (Word variations have been searched) | **3** |
| **Cochrane Database of Systematic Reviews**  http://onlinelibrary.wiley.com/cochranelibrary/search | (estrus OR oestrus OR estrous OR oestrous OR anestrus OR anoestrus OR anestrous OR anoestrous OR proestrus OR proestrus OR proestrous OR proestrous OR metestrus OR metoestrus OR metestrus OR metoestrous OR diestrus OR dioestrus OR diestrous OR dioestrous OR "ovarian cycle" OR ovulation OR "luteal phase" OR "uterine cycle" OR menstruation OR menses OR menstruating OR menstrual OR amenorrhea OR "proliferative phase" OR "secretory phase") AND ("tooth movement" OR "orthodontic movement" OR "orthodontic anchorage" OR root resorption) in Title Abstract Keyword - (Word variations have been searched) | **0** |
| **Scopus**  https://www.scopus.com | TITLE-ABS-KEY((estrus OR oestrus OR estrous OR oestrous OR anestrus OR anoestrus OR anestrous OR anoestrous OR proestrus OR proestrus OR proestrous OR proestrous OR metestrus OR metoestrus OR metestrus OR metoestrous OR diestrus OR dioestrus OR diestrous OR dioestrous OR "ovarian cycle" OR ovulation OR "luteal phase" OR "uterine cycle" OR menstruation OR menses OR menstruating OR menstrual OR amenorrhea OR "proliferative phase" OR "secretory phase") AND ("tooth movement" OR "orthodontic movement" OR "orthodontic anchorage" OR root resorption)) | **8** |
| **Web of Science™**  http://apps.webofknowledge.com/ | TOPIC: ((estrus OR oestrus OR estrous OR oestrous OR anestrus OR anoestrus OR anestrous OR anoestrous OR proestrus OR proestrus OR proestrous OR proestrous OR metestrus OR metoestrus OR metestrus OR metoestrous OR diestrus OR dioestrus OR diestrous OR dioestrous OR "ovarian cycle" OR ovulation OR "luteal phase" OR "uterine cycle" OR menstruation OR menses OR menstruating OR menstrual OR amenorrhea OR "proliferative phase" OR "secretory phase") AND ("tooth movement" OR "orthodontic movement" OR "orthodontic anchorage" OR root resorption)) Timespan: All years. Databases: WOS, KJD, RSCI, SCIELO, ZOOREC. Search language=Auto | **14** |
| **Arab World Research Source**  http://0-web.a.ebscohost.com.amclb.iii.com | TI tooth movement OR AB tooth movement | **4** |
| **ProQuest Dissertations and Theses Global**  http://search.proquest.com/dissertations | ti((estrus OR oestrus OR estrous OR oestrous OR anestrus OR anoestrus OR anestrous OR anoestrous OR proestrus OR proestrus OR proestrous OR proestrous OR metestrus OR metoestrus OR metestrus OR metoestrous OR diestrus OR dioestrus OR diestrous OR dioestrous OR "ovarian cycle" OR ovulation OR "luteal phase" OR "uterine cycle" OR menstruation OR menses OR menstruating OR menstrual OR amenorrhea OR "proliferative phase" OR "secretory phase") AND ("tooth movement" OR "orthodontic movement" OR "orthodontic anchorage" OR root resorption)) OR ab((estrus OR oestrus OR estrous OR oestrous OR anestrus OR anoestrus OR anestrous OR anoestrous OR proestrus OR proestrus OR proestrous OR proestrous OR metestrus OR metoestrus OR metestrus OR metoestrous OR diestrus OR dioestrus OR diestrous OR dioestrous OR "ovarian cycle" OR ovulation OR "luteal phase" OR "uterine cycle" OR menstruation OR menses OR menstruating OR menstrual OR amenorrhea OR "proliferative phase" OR "secretory phase") AND ("tooth movement" OR "orthodontic movement" OR "orthodontic anchorage" OR root resorption)) | **1** |

**Supplementary Table 3.** Quality of available evidence.

| - **Quality assessment** | | | | | | - **Effect direction** | - **Quality** |
| --- | --- | --- | --- | --- | --- | --- | --- |
| - **Studies** | - **Risk of bias** | - **Inconsistency** | - **Indirectness** | - **Imprecision** | - **Other** |  |  |
| **Difference in the amount of tooth movement between the various stages of oestrous cycle** | | | | | | | |
| - 3 | - Not serious | - Not serious | - Not serious | - Serious^1^ | - None | - More tooth movement when oestrogen levels are lower | - ⨁⨁⨁◯ - **MODERATE** |

- ^1^ The number of animals analysed was limited
